# Climate change redefines sea turtle hotspots: Vessel strike risks and gaps in protected areas

**DOI:** 10.1126/sciadv.adw4495

**Published:** 2025-06-25

**Authors:** Edouard Duquesne, Denis Fournier

**Affiliations:** Evolutionary Biology & Ecology, Université libre de Bruxelles (ULB), Av. F.D. Roosevelt, 50, CP 160/12, 1050 Brussels, Belgium.

## Abstract

Climate change is altering marine ecosystems, driving shifts in sea turtle distributions and challenging conservation efforts. Our study examines how climate change affects the global sea distribution of all seven sea turtle species, intersecting with marine protected areas (MPAs) and shipping corridors. Using species distribution models and environmental data from 2000 to 2024, we project sea turtle habitats under current conditions and three future climate scenarios (SSP1-2.6, SSP2-4.5, and SSP5-8.5) for 2050 and 2100. Our results show substantial habitat redistributions, with poleward shifts and contractions, particularly under the SSP5-8.5 scenario. Over 50% of sea turtle hotspots may disappear by 2050, with many new habitats in high shipping intensity areas. Alarmingly, only 23% of current hotspots are within MPAs, highlighting the need for adaptive conservation strategies.

## Teaser

As oceans warm, sea turtles flee the tropics, straight into the path of global maritime traffic.

## INTRODUCTION

Climate change affects all ecosystems and species on Earth, altering habitats, disrupting ecological balances, and affecting species survival across marine and terrestrial realms. With global temperatures rising at a rate outpacing species’ evolutionary adaptation, the resilience of natural systems is under unprecedented strain (*1*–*3*). This rapid temperature rise is predicted to disturb ecological assemblages dramatically, with up to 30% of species potentially surpassing thermal thresholds under 1.5° to 2.5°C warming scenarios (*4*, *5*). Current projections indicate a potential temperature rise of up to 3.1°C by the end of the century, over twice the target established nearly a decade ago (*6*).

One prominent biological response to warming is the poleward migration of species toward cooler climates, observed across both marine and terrestrial environments. This movement represents a complex ecological shift with broad implications for species interactions and ecosystem services that are fundamental to biodiversity and human well-being (*7*, *8*). Marine ectotherms, whose physiology is highly dependent on ambient temperatures, are especially vulnerable to climate-driven environmental changes (*3*, *9*). With fewer natural barriers than terrestrial organisms, marine species, in particular pelagic ones, have shown a broader poleward or deeper migration in search of suitable thermal habitats (*10*–*13*). However, such shifts may increase their risk of entering high-traffic maritime corridors, exposing these species to vessel strikes, bycatch, and other anthropogenic impacts (*14*, *15*).

Large marine vertebrates such as whales, sharks, dolphins, porpoises, whale sharks, seals, and sea turtles, are particularly vulnerable to these anthropogenic threats, which can threaten their populations and have cascading effects on marine ecosystems (*15*, *16*). Shifting species distributions further highlight limitations in conventional marine conservation strategies, as many marine protected areas (MPAs), whether in national water or high seas as registered by the World Database on Protected Areas, are static and unable to address dynamic species distributions (*17*, *18*). Effective conservation now requires adaptive strategies that identify and protect critical habitats throughout the annual cycles of migratory species, while also fostering an understanding of ecological connectivity across regions (*19*). This urgency aligns with the Kunming-Montreal Global Biodiversity Framework’s goal to protect 30% of marine areas by 2030 (*20*).

Dynamic ocean management, which adjusts protection based on real-time environmental and species distribution data, has emerged as a promising approach to align conservation efforts with the shifting ranges of marine species. By integrating data on environmental conditions, species movement, and human activities, this approach can help mitigate risks related to bycatch and vessel strikes, thereby reducing human-related impacts on vulnerable marine species (*21*–*23*).

Among marine species, sea turtles hold a critical ecological role as keystone species in ocean ecosystems (*24*). Yet, six of the seven marine turtle species are classified as vulnerable, endangered or critically endangered on the International Union for Conservation of Nature (IUCN) Red List (one species is classified as “data deficient”), primarily due to human-driven pressures like pollution, coastal development, and overfishing (*25*–*28*). Climate change compounds these existing threats by altering the temperature gradients and ocean currents that guide turtles’ long-distance movements between nesting and foraging areas (*29*–*33*).

With global warming, sea turtles are likely to encounter serious challenges in both their terrestrial and marine habitats. Rising temperatures threaten to skew sex ratios toward feminization of populations and reduce suitable nesting grounds, with models indicating substantial habitat loss within both short-term (2021–2040) and medium-term (2041–2060) projections (*34*, *35*). These environmental changes may prompt shifts in turtles’ nesting and foraging areas (*34*, *36*–*38*), increasing their exposure to high-risk maritime zones where vessel traffic is dense or in regions not covered by MPAs. This vulnerability is exacerbated by the rapid expansion of maritime activity. Recreational boating has expanded at an average of 10% annually since 2008 in Mediterranean Europe and is projected to grow globally at a compound annual rate of 5.1% from 2025 to 2030 (*39*, *40*). In parallel, maritime trade, responsible for over 90% of global goods transport, has quadrupled since 1992 (*41*, *42*). Vessel strikes are now a major cause of sea turtle mortality in regions with intensive marine traffic, such as the Galapagos Islands, Hawaiian Archipelago, Malaysian coasts, the Mediterranean Sea, and U.S. Atlantic waters [see (*43*) and references therein]. In Florida alone, vessel strikes are the leading identifiable cause of sea turtle death (*44*).

Using a dataset of 27,703 filtered sea turtle occurrences and more than 1 billion Automatic Identification System (AIS) vessel locations (commercial, fishing, passenger, oil and gas, and recreational ships), our study investigates how climate change may influence sea turtle migration patterns and assesses the ensuing collision risks posed by maritime traffic. Using high-resolution species distribution models integrated with advanced vessel detection data, we projected future habitat suitability for sea turtles under three shared socioeconomic pathways (SSP1-2.6, SSP2-4.5, and SSP5-8.5) for both mid-century (2050) and end-of-century (2100) timeframes. Developed for the Intergovernmental Panel on Climate Change (IPCC) Sixth Assessment Report (2021), these pathways represent divergent global trajectories, from sustainable development (SSP1), to intermediate trends (SSP2), to fossil-fueled development (SSP5) (*45*). By analyzing the spatial overlap between predicted sea turtle distributions, MPAs, and vessel traffic, we identified high-risk zones requiring focused conservation efforts.

## RESULTS

Both individual and ensemble models demonstrated strong predictive performance, with ensemble averages achieving high values for both the receiver operating characteristic (ROC; 0.993) and true skill statistic (TSS; 0.935) metrics (see table S7 for details). Key contributing variables included temperature (air or ocean), bathymetry, primary productivity and dissolved oxygen (table S8). Summary figures for each species, including MPAs and shipping intensity, are provided in figs. S1 to S7.

### Current and future conditions of sea turtles

**Fig. 1.**
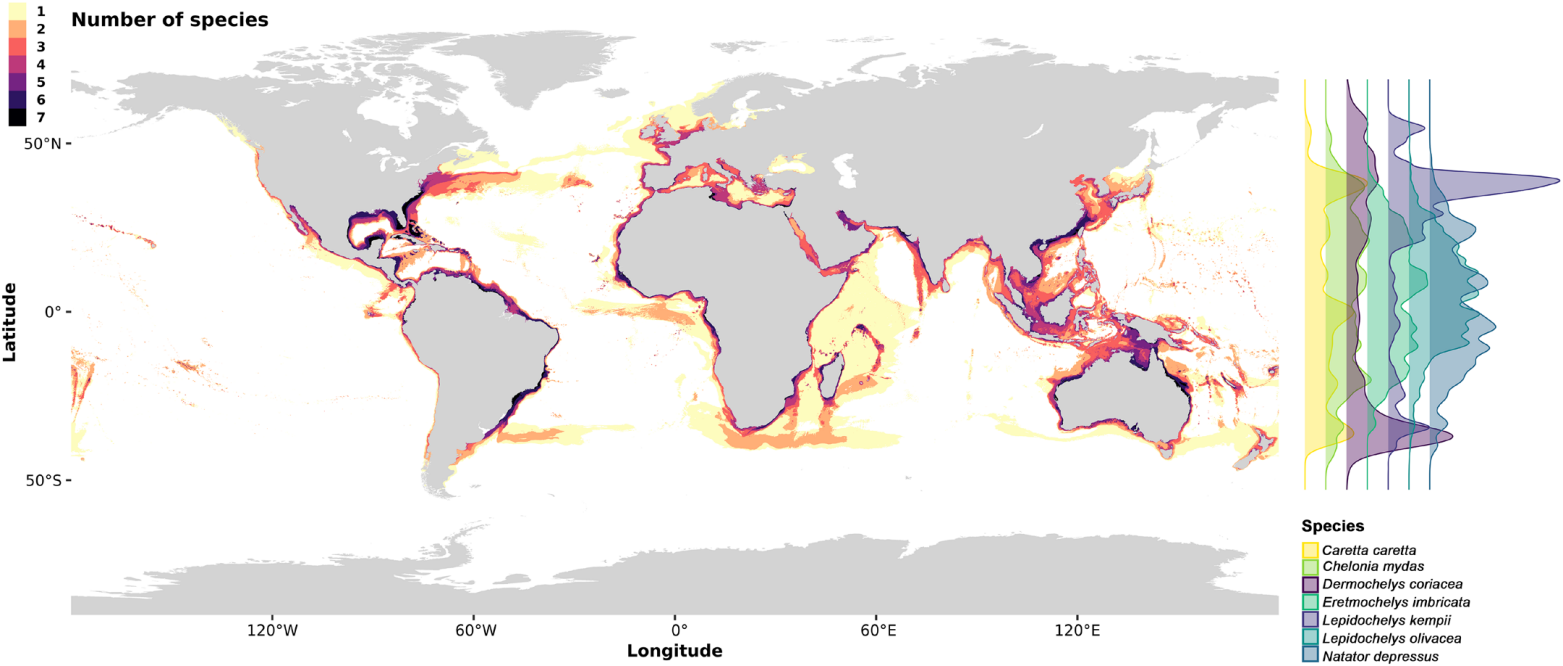
Current species distribution and latitudinal density for each species. Number of species currently suitable globally (**left**) and latitudinal density for each species (**right**).

Our analysis reveals that sea turtles currently occupy extensive suitable habitats across global oceans, with several overlapping hotspots supporting multiple species concurrently. Notable critical areas include the coasts of Australia, the eastern United States, Mexico, South America, and eastern Asia. These regions host the highest species densities, with most species exhibiting broad tropical distributions. Notably, *Caretta caretta* (Linnaeus, 1758), *Dermochelys coriacea* (Vandelli, 1761), and *Lepidochelys kempii* (Garman, 1880) extend into higher latitudes (Fig. 1).

Future projections under three shared socioeconomic pathways (SSP1-2.6, SSP2-4.5, and SSP5-8.5) indicate substantial variability among species. Four sea turtle species, *C. caretta*, *L. kempii*, *Lepidochelys olivacea* (Eschscholtz, 1829), and *D. coriacea*, are projected to undergo substantial range contractions, with predicted reductions averaging between 17 and 67% across all scenarios and timeframes. Conversely, three species [*Chelonia mydas* (Linnaeus, 1758), *Eretmochelys imbricata* (Linnaeus, 1766), and *Natator depressus* (Garman, 1880)] show potential range expansions, averaging 23 to 34% (table S6). These shifts suggest a pronounced redistribution of sea turtle habitats due to global climate change, with varying impacts depending on latitude and hemisphere.

**Fig. 2.**
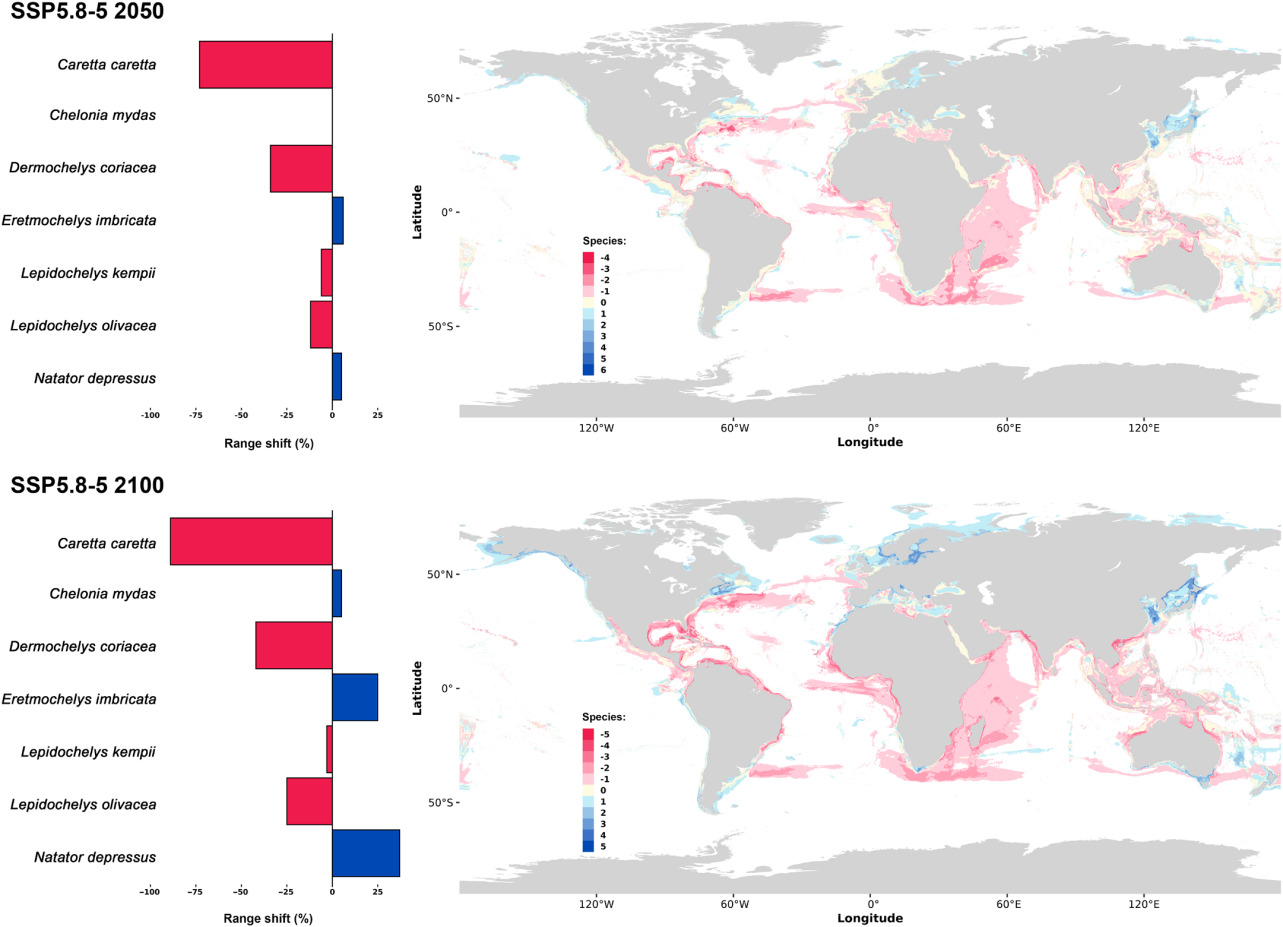
Range shifts and net pixel change. Range shifts (net pixel gain or loss) in sea turtle habitats between current conditions and projections under the SSP5-8.5 scenario for 2050 (top left) and 2100 (bottom left) for each species. The right panels show the number of species gained (blue) or lost (red) per pixel by 2050 (top right) and 2100 (bottom right). Pixels with an equal number of species gained and lost are shown in light yellow.

Under the SSP5-8.5 scenario, the most extreme pathway, vast tropical regions, in particular in the southern hemisphere, become less suitable for most species by 2100. This trend intensifies compared to mid-century projections, indicating a notable northward and southward shift toward cooler regions. Specifically, the Baltic Sea, East China Sea, Sea of Japan, and the Canadian Atlantic are anticipated to see increased sea turtle presence in the northern hemisphere. Similar shifts occur in the southern hemisphere around South Africa, southern South America, the Bass Detroit, and the Tasman Sea (Fig. 2).

### Sea turtles’ hotspots, MPAs, and exclusive economic zones

Current models estimate that sea turtle hotspots, regions with high suitability for multiple species, encompass approximately 2 million square kilometers of seas and oceans, with 89% of these areas located within exclusive economic zones (EEZs; maritime zones extending from a nation’s coastline where it holds exclusive rights to resource use). Alarmingly, only 23% of these critical habitats fall within MPAs, primarily due to important contributions from Australia. Conversely, many EEZs, including those of the United States and China, protect less than 10% of their identified hotspots (6.3 and 0.2%, respectively) despite hosting substantial areas of critical habitat. Regionally, most hotspots are currently located in the North Atlantic Ocean (766,243 km^2^), followed by the Indian Ocean (365,810 km^2^), the South Pacific Ocean (220,608 km^2^), the South China and Eastern Archipelagic Seas (210,997 km^2^), and the South Atlantic Ocean (173,295 km^2^), each with varying levels of protection: 11.4, 21.3, 83.2, 2.34, and 24.6%, respectively (table S9).

**Fig. 3.**
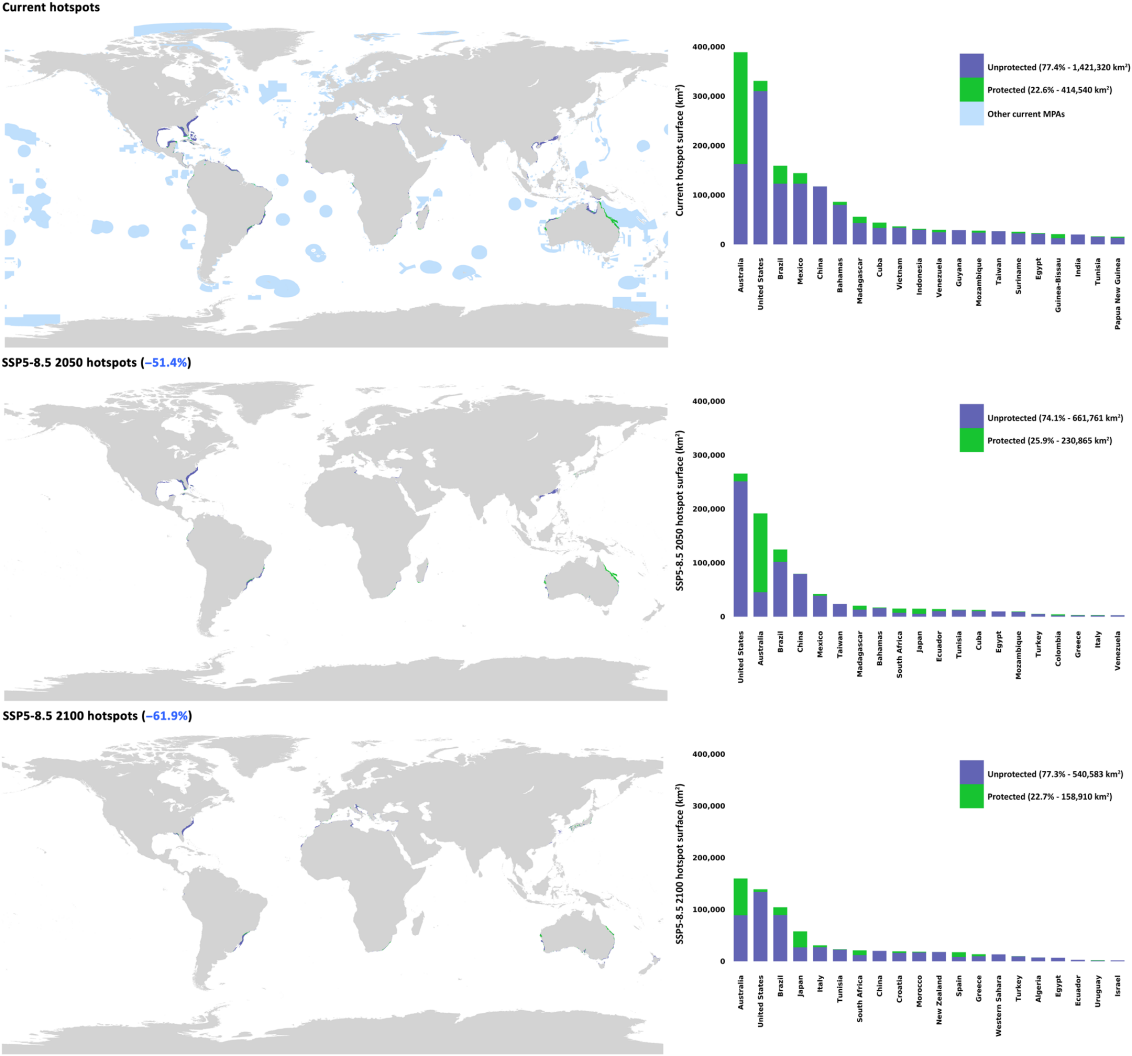
Current and projected sea turtle hotspots under SSP5-8.5 for 2050 and 2100 and hotspots areas within EEZs. On the left, current and future sea turtle hotspots are shown, categorized as marine protected hotspots (green), unprotected hotspots (purple), and, for the current scenario only, other MPAs not overlapping with hotspots (blue). On the right the distribution of protected and unprotected hotspot areas within the exclusive economic zones (EEZs) of the 20 countries with the largest hotspot surfaces is shown.

Future projections under the SSP5-8.5 scenario forecast a marked reduction in hotspot areas, shrinking by over 50% by 2050 (to around 900,000 km^2^, with 97% being in EEZ) and more than 60% by 2100 (to around 700,000 km^2^, with 98% being in EEZ). The proportion of protected versus unprotected hotspots remains relatively stable, ranging between 26 and 23% across projected timelines (Fig. 3). For the details about regional projections, refer to table S9.

### Projected sea turtle distributions and shipping intensity

**Fig. 4.**
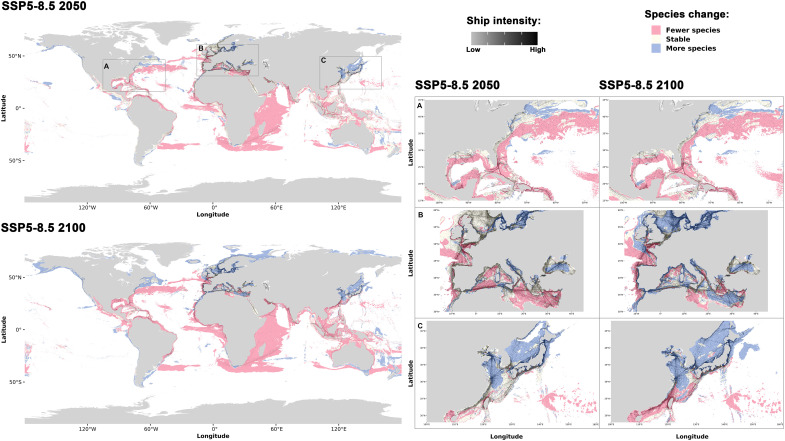
Species distribution changes between current conditions and SSP5-8.5 for 2050 and 2100. Changes in terms of species distribution are shown (red = decline, yellow = stable, and blue = increase). Shipping intensity is indicated with a black scale. Enlarged insets highlight key regions: Eastern North America (**A**), Europe (**B**), and Eastern Asia (**C**).

Our assessment of projected sea turtle distributions under the SSP5-8.5 scenario, combined with current vessel traffic data, reveals substantial risks associated with habitat suitability and shipping intensity. Regions projected to support fewer species generally coincide with low shipping traffic areas. In contrast, areas with high shipping activity, such as Europe’s North Sea and Mediterranean Sea as well as Asia’s East China Sea and Sea of Japan, are expected to become more suitable for several sea turtle species by 2100 (Fig. 4).

Eastern North America, characterized by relatively low shipping intensity compared to Europe and Asia, shows a pronounced decline in species numbers, with some stabilization near the coasts. This trend worsens with longer timeframes. In contrast, European waters, in particular those with high shipping density (the North Sea and the Mediterranean Sea), may experience increased turtle presence, raising concerns about heightened collision risks and habitat disturbances. Similar patterns emerge in Eastern Asia, where the East China Sea, Yellow Sea, and Sea of Japan, regions of escalating shipping activity, are projected to host several species (Fig. 4).

## DISCUSSION

This study provides crucial insights into the projected shifts in the global distribution of all seven sea turtle species under various climate change scenarios, emphasizing the far-reaching implications for conservation strategies and marine policy frameworks. By comparing current and future distributions with existing MPAs and shipping routes, we highlight emerging challenges for these already vulnerable keystone species.

### Shifts in habitats and migration patterns

Our findings align with broader trends in marine biodiversity redistribution under climate change. As fish stocks are predicted to shift toward higher latitudes, tropical regions are transforming from biodiversity sinks to sources, while temperate and polar regions increasingly become new biodiversity reservoirs (*10*, *46*, *47*). This phenomenon, driven by rising ocean temperatures and changing currents, is expected to impact not only fisheries (*48*), but also migratory species like sea turtles. Consistent with prior studies on their sensitivity to climate-driven habitat alterations (*36*, *49*, *50*), our results suggest that sea turtles will exhibit poleward shifts, with tropical and subtropical areas becoming increasingly unsuitable. Under the SSP5-8.5, toward which current global trajectories appear to be heading (*6*), some species could experience important range contractions, posing severe conservation challenges given their current endangered status (*28*).

### Challenges for MPAs

The anticipated range shifts raise critical concerns about the efficacy of existing MPAs. Designed under the assumption of relatively stable environmental conditions (*51*), many MPAs may no longer overlap with future sea turtle habitats. Although MPAs provide vital refuges from human threats (*17*), their role in climate change adaptation has largely been conceptual rather than operational (*52*). Our findings indicate that under SSP5-8.5, sea turtle hotspots, areas highly suitable for multiple species, could decline by up to 50% by 2050 and 2100, both within and outside MPAs. This reduction suggests that without dynamic, adaptive management strategies, current MPAs may become less effective, potentially leaving sea turtles more vulnerable as they migrate beyond protected boundaries (*34*, *38*).

### Increasing risk from vessel strikes

Among the most pressing anthropogenic threats, vessel strikes have been well-documented globally, particularly in shallow coastal waters where both boat traffic and sea turtle densities are high (*43*, *44*, *53*–*56*). Our projections indicate an exacerbation of this threat as sea turtles migrate toward regions with some of the highest global shipping intensities, such as the North Sea, the Mediterranean and the East China Sea. Given that global shipping activity is expected to increase markedly, by 240% to 1209% by 2050 (*57*), the risk of vessel strikes will likely escalate, mirroring patterns observed in other wide-ranging marine species like whale sharks and whales (*58*, *59*). Parallel increases in recreational boating activity (*39*, *40*) and the extension of boating season under warmer conditions further elevate this risk. Implementing vessel speed reductions in identified hotspot areas presents a practical and effective mitigation strategy, as slower speeds have been shown to markedly reduce the likelihood and severity of collisions (*56*, *60*).

### Broader ecological and societal impacts

The compounded threats of climate change and vessel strikes on sea turtles extend beyond species-level concerns. As keystone species, sea turtles play critical roles in maintaining healthy marine ecosystems, including regulating seagrass beds and contributing to nutrient cycling (*24*). Their decline could disrupt these ecosystems, with cascading effects on biodiversity and human livelihoods (*61*). Protecting sea turtles is therefore not only a conservation priority but also essential for sustaining ecosystem services upon which coastal communities depend. Effectively addressing these multifaceted challenges requires an integrated, adaptive approach to marine conservation. Enhancing the resilience of MPAs through dynamic boundaries and climate-informed management, coupled with stringent regulation of shipping in critical habitats, is imperative. International collaboration, robust policy frameworks, and real-time monitoring systems will be key to safeguarding sea turtles and the complex marine ecosystems they support.

## MATERIALS AND METHODS

### Experimental design

Our analytical framework comprised seven key steps: (i) compiling and cleaning raw species occurrence data from 2000 to 2024, (ii) selecting relevant environmental variables for habitat suitability modeling, (iii) mapping vessel traffic density and MPAs, (iv) generating habitat suitability models for the current and future conditions, (v) assessing sea turtle habitats under present and projected conditions, (vi) identifying hotspots within MPAs, and (vii) estimating co-occurrence with shipping routes under various climate projections.

### Species occurrence data

We downloaded occurrence data from the Global Biodiversity Information Facility (*62*–*69*) for all seven sea turtle species and processed it using R version 4.1.2 (*70*) with packages dplyr 1.1.4 (*71*) and sf 1.0.18 (*72*). To ensure data quality, we removed duplicate and unprecise coordinates using dplyr and the CoordinateCleaner 3.0.1 package to filter suspicious occurrences [e.g., identical longitude and latitude, Global Biodiversity Information Facility (GBIF) headquarters…] (*73*). We excluded terrestrial occurrences based on geographic polygons corresponding to countries and dependent territories (ISO 3166-1 alpha-3) from the rnaturalearth 1.0.1 package (*74*). We applied spatial thinning (5.5 km minimum distance, resolution of the models) using spThin 0.2.0 (*75*) to reduce sampling biases. GBIF DOIs and the number of occurrences before and after filtering are available in table S1. Final occurrence data (ranging from 342 to 13,884 occurrences per species) are available in data S1, while the ODMAP (Overview, Data, Model, Assessment, and Prediction) protocol is available in table S2 (*76*).

### Environmental variables

Explanatory variables were downloaded from Bio-ORACLE version 3 in 0.05 resolution (±5.5 km grid) (*77*, *78*), comprising 22 global marine layers capturing physical, chemical, biological, and topographic factors essential to sea turtle habitat (table S3). Present-day layers spanned 2000 to 2020 [grid-cell mean computed using QGIS 3.34.11 (*79*)], while future projections were derived from the Coupled Model Intercomparison Project 6 framework under three Shared Socioeconomic Pathways (SSPs) for mid- (2050) and long-term (2100) scenarios. These included: (i) a “sustainability” scenario (SSP1-2.6), characterized by a global shift toward inclusive development and human well-being over economic growth, with improved management of the global commons, reduced inequality, declining resource and energy use, net-zero CO_2_ emissions around or after 2050, and warming limited to ~1.8°C by the end of the century; (ii) a “middle of the road” scenario (SSP2-4.5), extrapolating historical and development trends and resulting in an estimated 2.7°C warming by 2100; and (iii) a “fossil-fueled development” scenario (SSP5-8.5), reflecting a future driven by intensive fossil fuel use, with emissions projected to double by 2050 and warming reaching ~4.4°C in 2100. To prevent overfitting, we retained the top six noncorrelated (*r* < 0.70) variables for each species. The selection process was done using the R package Biomod2 4.2.4 (*80*) with the variable importance function using ten different algorithms (MAXENT, GAM, GLM, ANN, CTA, MARS, GBM, SRE, XGBOOST, and RF). Except for MAXENT and XGBOOST, for which we used default options, the algorithms were parameterized based on the recommended options given in the “big boss” strategy from the package developers (version 4.2.5). Pearson correlation and variable importance are provided in tables S4 and S5.

### Vessel traffic data, MPAs, and EEZs

We evaluated the risks of vessel impacts on sea turtles using global vessel detection data from (*81*), which are derived from radio signals emitted by ships for navigational safety. This dataset integrates signals from commercial, fishing, oil and gas, passenger and recreational vessels, and applies machine-learning algorithms to estimate shipping activity intensity from January 2015 to February 2021 (*81*). To highlight high density traffic areas, the raster data were transformed using a square root scale. For visual clarity in the main figures, only pixels above the mean density were retained.

Data on MPAs (January 2025) were downloaded from Protected Planet (*82*). Vector files were rasterized using the terra package [version 1.7.78; (*83*)] and overlaid on the habitat suitability maps. Vector data on EEZs and global oceans and seas were downloaded from Marine Regions website [https://marineregions.org/, (*84*, *85*)].

### Species distribution modeling

For each species, we randomly generated 5000 pseudoabsences with three repetitions using Biomod2 (*86*). Modeling and evaluation were conducted with the same 10 algorithms and parameters used during variable selection. To train the models, we randomly selected 75% of the occurrence data, reserving the remaining 25% for evaluation using the TSS (*87*) and ROC [or area under the curve (*88*)]. Models were validated using 5 + 1–fold cross-validation for each species. Ensemble models were generated using a weighted average of high-TSS models (>0.75) (*89*) and projected onto future climate scenarios using selected environmental layers. The ensemble projections (habitat suitability maps) were also converted into binary maps based on optimized TSS thresholds through the Biomod2 package.

For current conditions, binary maps for the seven species were aggregated to identify areas with high suitability for sea turtles. We also calculated latitudinal density for each species to determine their optimal latitudinal ranges. Under future scenarios (SSP1-2.6, SSP2-4.5, and SSP5-8.5), range shifts were quantified using Biomod2’s built-in functions to estimate the number of pixels lost, stable, or gained between current and projected conditions (table S6). For the SSP5-8.5 scenario, we summed the range changes of all seven species to produce a map showing the net gain or loss in species richness per pixel.

To identify current and future sea turtle hotspots, we summed the suitability maps of each species before standardizing the resulting raster (0 to 1 scale). Only areas with a suitability score greater than 0.80 were retained as hotspots. These hotspots were then overlaid with MPA boundaries to assess conservation coverage, identifying both protected and unprotected critical areas. We further analyzed EEZs to highlight conservation priorities at a national level.

Last, we evaluated the impacts of shipping on sea turtles by overlaying vessel traffic density maps with species distribution changes between current conditions and the SSP5-8.5 scenario.
