# Supplementary material for: Climate change redefines sea turtle hotspots: Vessel strike risks and gaps in protected areas
Source: Sci Adv. 2025 Jun 25;11(26):eadw4495. doi: 10.1126/sciadv.adw4495 (PMC12189953; doi:10.1126/sciadv.adw4495)
Supplement: Supplementary file 1 — Figs. S1 to S7 Tables S1 to S9 [file sciadv.adw4495_sm.pdf]

Science Advances
AAAS

Supplementary Materials for

Climate change redefines sea turtle hotspots: Vessel strike risks and gaps in protected areas

Edouard Duquesne and Denis Fournier

Corresponding author: Edouard Duquesne, edouard.duquesne@ulb.be; Denis Fournier, Denis.Fournier@ulb.be

Sci. Adv. **11**, eadw4495 (2025)
DOI: 10.1126/sciadv.adw4495

This PDF file includes:

Figs. S1 to S7
Tables S1 to S9

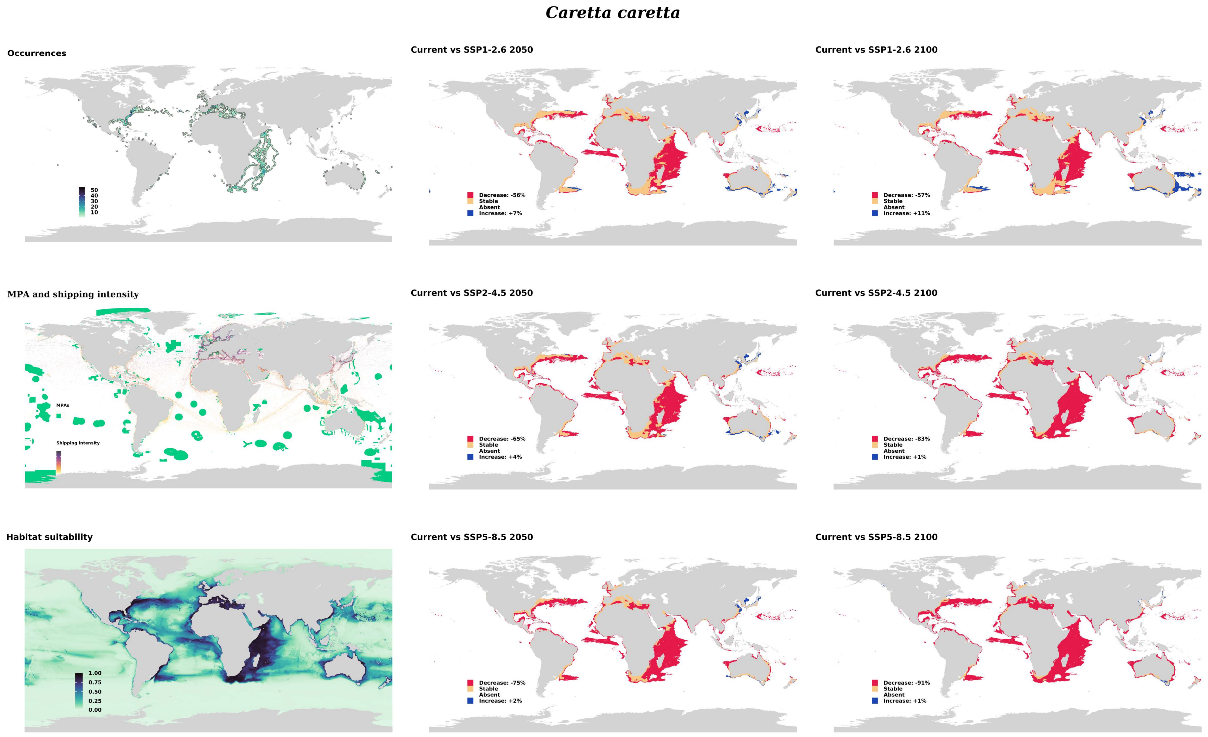

fig. S1. Comprehensive results for *Caretta caretta* habitat and range dynamics.

From top left to bottom right, arranged vertically: (1) number and spatial distribution of occurrences; (2) marine protected areas (green) and shipping intensity; (3) current habitat suitability; (4) projected range changes under SSP1-2.6 for 2050; (5) projected range changes under SSP2-4.5 for 2050; (6) projected range changes under SSP5-8.5 for 2050; (7) projected range changes under SSP1-2.6 for 2100; (8) projected range changes under SSP2-4.5 for 2100; and (9) projected range changes under SSP5-8.5 for 2100.

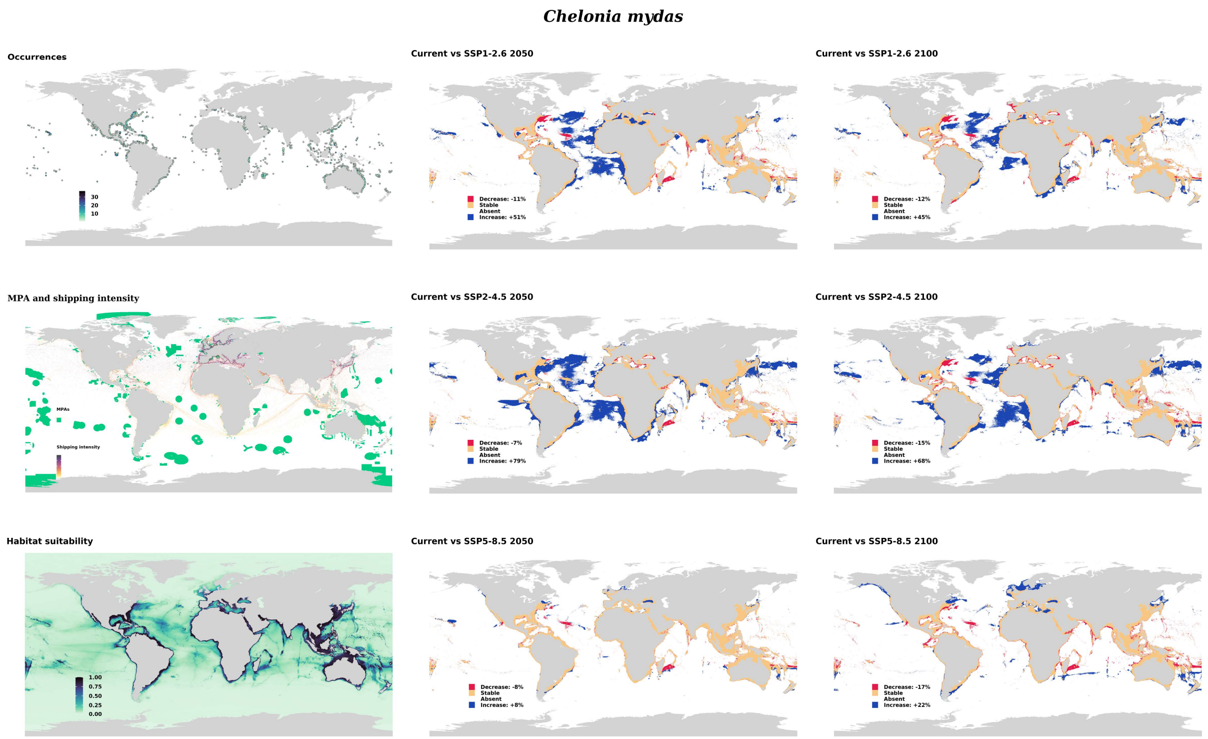

fig. S2. Comprehensive results for *Chelonia mydas* habitat and range dynamics.

From top left to bottom right, arranged vertically: (1) number and spatial distribution of occurrences; (2) marine protected areas (green) and shipping intensity; (3) current habitat suitability; (4) projected range changes under SSP1-2.6 for 2050; (5) projected range changes under SSP2-4.5 for 2050; (6) projected range changes under SSP5-8.5 for 2050; (7) projected range changes under SSP1-2.6 for 2100; (8) projected range changes under SSP2-4.5 for 2100; and (9) projected range changes under SSP5-8.5 for 2100.

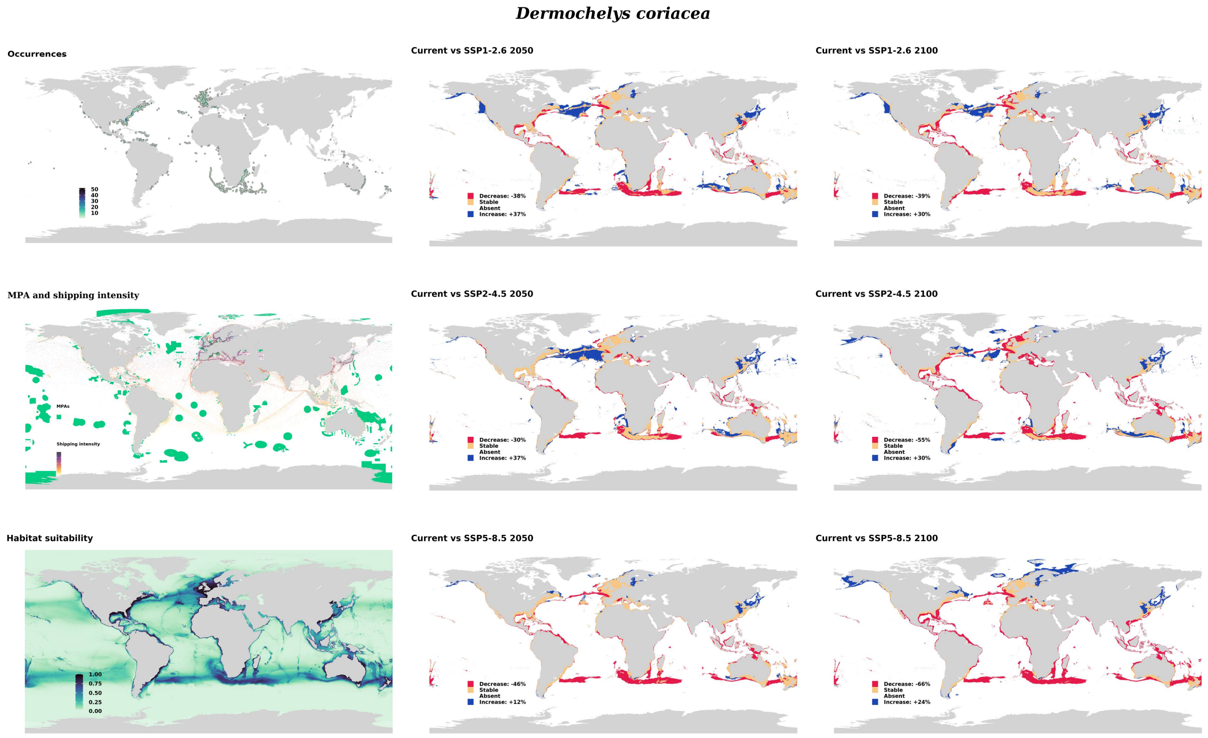

fig. S3. Comprehensive results for *Dermochelys coriacea* habitat and range dynamics.

From top left to bottom right, arranged vertically: (1) number and spatial distribution of occurrences; (2) marine protected areas (green) and shipping intensity; (3) current habitat suitability; (4) projected range changes under SSP1-2.6 for 2050; (5) projected range changes under SSP2-4.5 for 2050; (6) projected range changes under SSP5-8.5 for 2050; (7) projected range changes under SSP1-2.6 for 2100; (8) projected range changes under SSP2-4.5 for 2100; and (9) projected range changes under SSP5-8.5 for 2100.

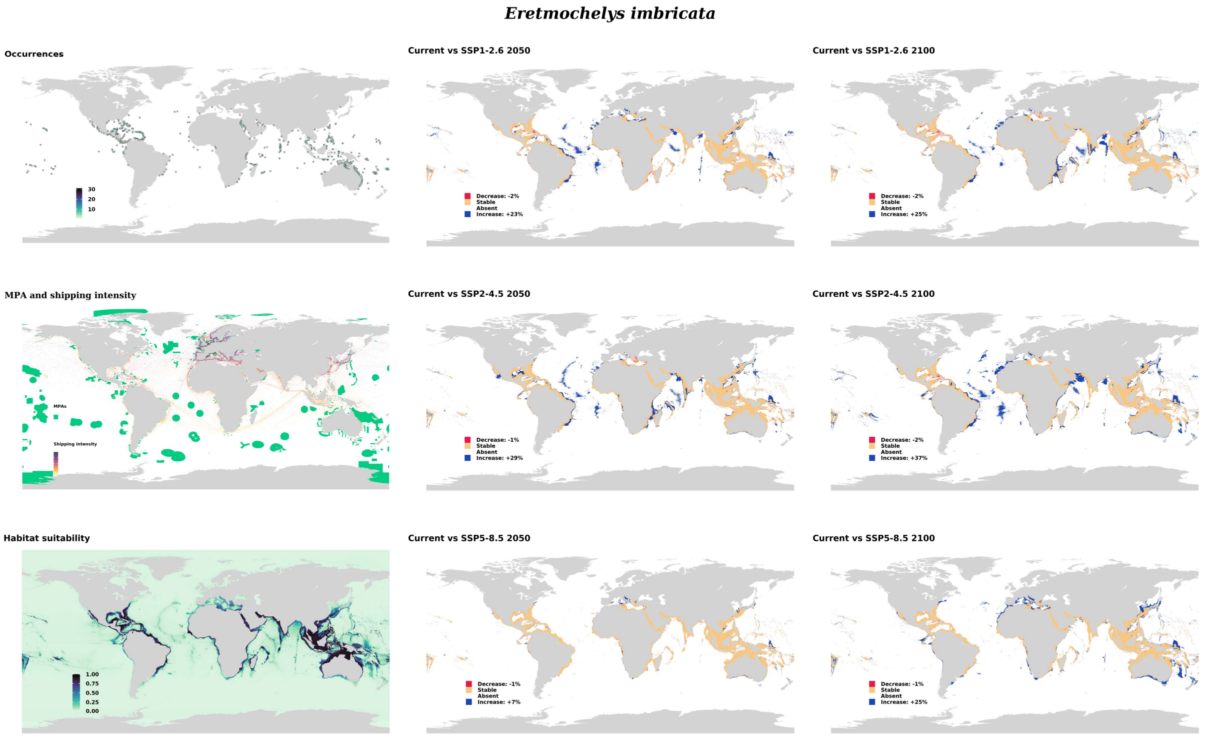

fig. S4. Comprehensive results for *Eretmochelys imbricata* habitat and range dynamics. From top left to bottom right, arranged vertically: (1) number and spatial distribution of occurrences; (2) marine protected areas (green) and shipping intensity; (3) current habitat suitability; (4) projected range changes under SSP1-2.6 for 2050; (5) projected range changes under SSP2-4.5 for 2050; (6) projected range changes under SSP5-8.5 for 2050; (7) projected range changes under SSP1-2.6 for 2100; (8) projected range changes under SSP2-4.5 for 2100; and (9) projected range changes under SSP5-8.5 for 2100.

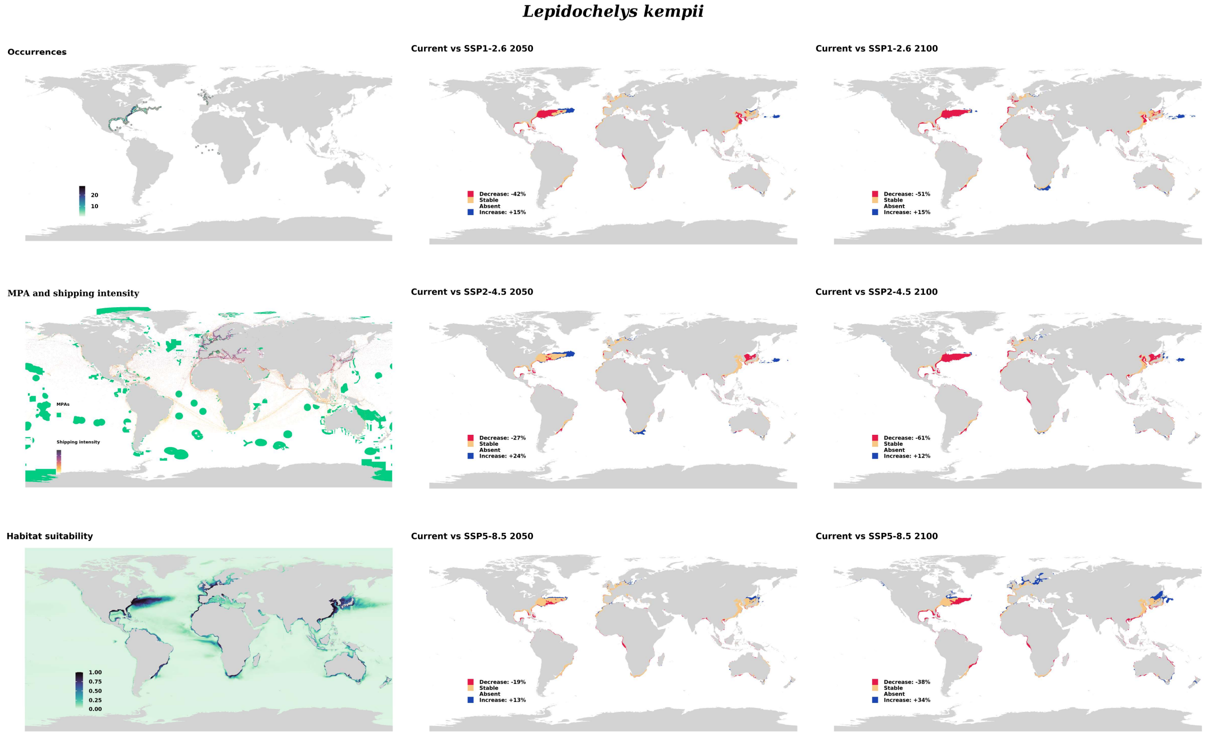

fig. S5. Comprehensive results for *Lepidochelys kempii* habitat and range dynamics.

From top left to bottom right, arranged vertically: (1) number and spatial distribution of occurrences; (2) marine protected areas (green) and shipping intensity; (3) current habitat suitability; (4) projected range changes under SSP1-2.6 for 2050; (5) projected range changes under SSP2-4.5 for 2050; (6) projected range changes under SSP5-8.5 for 2050; (7) projected range changes under SSP1-2.6 for 2100; (8) projected range changes under SSP2-4.5 for 2100; and (9) projected range changes under SSP5-8.5 for 2100.

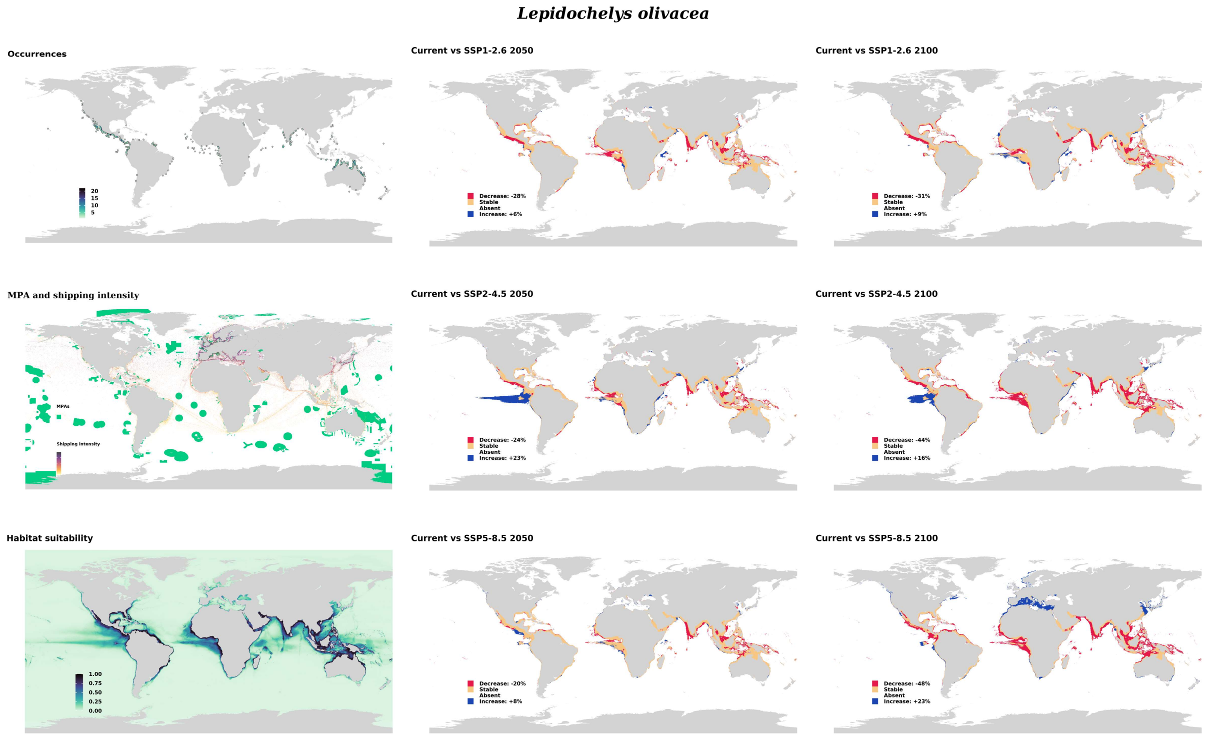

fig. S6. Comprehensive results for *Lepidochelys olivacea* habitat and range dynamics.

From top left to bottom right, arranged vertically: (1) number and spatial distribution of occurrences; (2) marine protected areas (green) and shipping intensity; (3) current habitat suitability; (4) projected range changes under SSP1-2.6 for 2050; (5) projected range changes under SSP2-4.5 for 2050; (6) projected range changes under SSP5-8.5 for 2050; (7) projected range changes under SSP1-2.6 for 2100; (8) projected range changes under SSP2-4.5 for 2100; and (9) projected range changes under SSP5-8.5 for 2100.

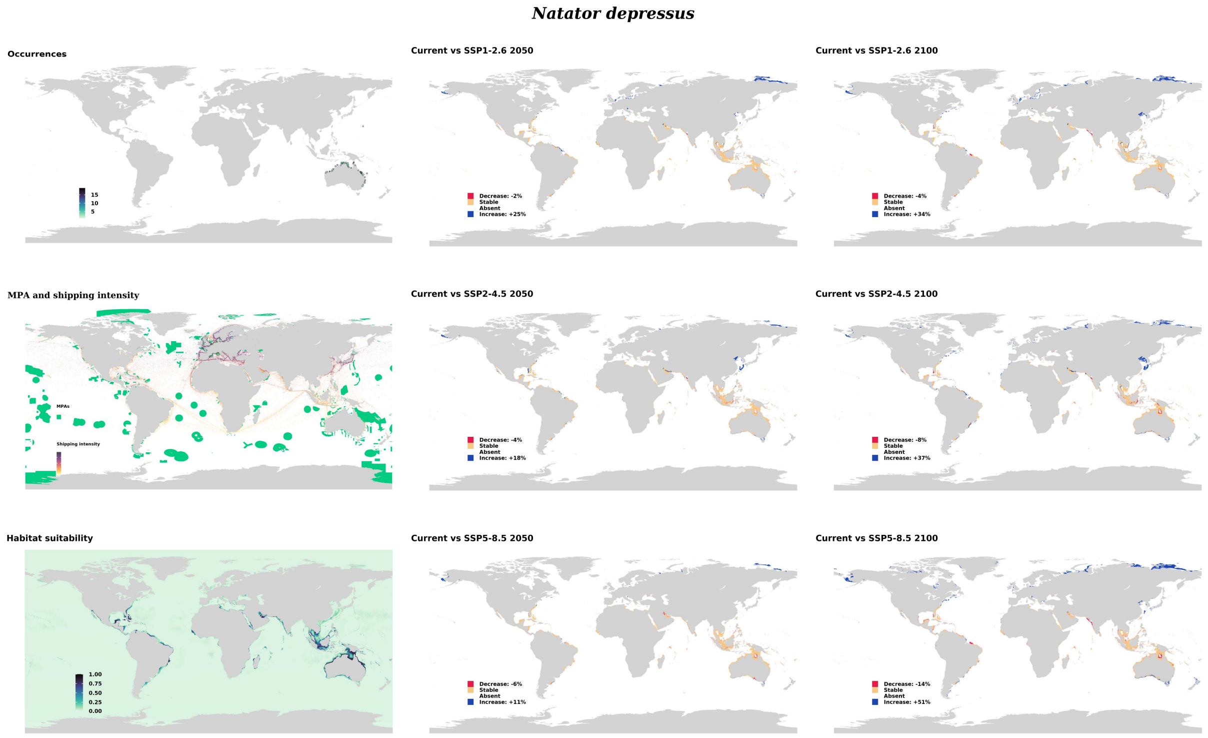

fig. S7. Comprehensive results for *Natator depressus* habitat and range dynamics.

From top left to bottom right, arranged vertically: (1) number and spatial distribution of occurrences; (2) marine protected areas (green) and shipping intensity; (3) current habitat suitability; (4) projected range changes under SSP1-2.6 for 2050; (5) projected range changes under SSP2-4.5 for 2050; (6) projected range changes under SSP5-8.5 for 2050; (7) projected range changes under SSP1-2.6 for 2100; (8) projected range changes under SSP2-4.5 for 2100; and (9) projected range changes under SSP5-8.5 for 2100.

table S1. Sources and number of occurrences analyzed in this study, including the raw data and the final dataset after filtering.

Details are provided in Data S1.

Source	Details	Occurrences (raw)	Occurrences (filtered)	Reference
GBIF	*Caretta caretta* (Linnaeus, 1758)	140685	13729	(*62*)
GBIF	*Chelonia mydas* (Linnaeus, 1758)	210626	5473	(*66*)
GBIF	*Dermochelys coriacea* (Vandelli, 1761)	19993	3082	(*67*)
GBIF	*Eretmochelys imbricata* (Linnaeus, 1766)	18592	2289	(*65*)
GBIF	*Lepidochelys kempii* (Garman, 1880)	4903	1365	(*64*)
GBIF	*Lepidochelys olivacea* (Eschscholtz, 1829)	24593	1497	(*63*)
GBIF	*Natator depressus* (Garman, 1880)	2631	268	(*61*)

table S2. Overview, Data, Model, Assessment and Prediction (ODMAP, (74)).

Section	Subsection	Element	Value
Overview	Authorship	Study title	Climate change redefines sea turtle hotspots: vessel strike risks and gaps in protected areas
Overview	Authorship	Author names	Duquesne Edouard and Fournier Denis
Overview	Authorship	Contact	edouard.duquesne@ulb.be, Denis.Fournier@ulb.be
Overview	Authorship	Study link	See paper
Overview	Model objective	Model objective	Predict habitat suitability of sea turtles in the ocean and co-occurrences with shipping
Overview	Model objective	Target output	Global ocean geographical map of predicted habitat suitability
Overview	Focal Taxon	Focal Taxon	Chelonioidea
Overview	Location	Location	Global
Overview	Scale of Analysis	Spatial extent	-180, 180, -90, 90 (xmin, xmax, ymin, ymax)
Overview	Scale of Analysis	Spatial resolution	~ 5.5 km²
Overview	Scale of Analysis	Temporal extent	Marine data layers: Decadal from present-day to the end of the 21st century (2000–2100), Occurrences 2000-2024
Overview	Scale of Analysis	Temporal resolution	Mean
Overview	Scale of Analysis	Boundary	lon/lat WGS 84 (EPSG:4326)
Overview	Biodiversity data	Observation type	standardized monitoring data, field survey, citizen science
Overview	Biodiversity data	Response data type	Presence-only
Overview	Predictors	Predictor types	Marine data layers
Overview	Hypotheses	Hypotheses	We test whether sea turtles will change their migratory journeys due to climate change and whether it could have an impact on co-occurrences with shipping
Overview	Assumptions	Model assumptions	We assume that available occurrence data are representative of species' contemporary distributions
Overview	Algorithms	Modelling techniques	SDM: BIOMOD2 (80) with the following algorithms: "MAXNET", "GAM", "GLM", "ANN", "CTA", "MARS", "GBM", "SRE", "XGBOOST", "RF"
Overview	Algorithms	Model complexity	Default settings for MAXNET and XGBOOST. The other algorithms were optimized using the recommended options with the "Bigboss" pre-defined parameter values for single models.
Overview	Algorithms	Model averaging	Ensemble modelling built from the individual models' weight using a sensitivity metric with a 0.75 threshold
Overview	Workflow	Model workflow	(1) Compile data from GBIF. (2) Clean the data. (3) Choose the best marine data layers variables using variable importance function from biomod2. (4) Generate 5000 pseudo-absences per species using the SDM package. (5) Randoms 75/25 splits for training and evaluation with five-cross validations. (6) Ensemble modelling for each species based on the individual models' weight using a sensitivity metric (0.75 threshold). (7) Projection of the model for the current conditions and for the different shared socioeconomic pathways.
Overview	Software	Software	R version 4.1.2. Terra package 1.7.78 (83) BIOMOD2 package 4.2-4 (80)
Overview	Software	Code availability	https://doi.org/10.5281/zenodo.15295865
Overview	Software	Data availability	https://doi.org/10.5281/zenodo.15295865
Data	Biodiversity data	Taxon names	Chelonioidea

Data	Biodiversity data	Taxonomic reference system	The Integrated Taxonomic Information System, DOI: 10.15468/kdu5gg
Data	Biodiversity data	Ecological level	species
Data	Biodiversity data	Data sources	GBIF (63-69), see table S1 for more information
Data	Biodiversity data	Sampling design	NA
Data	Biodiversity data	Sample size	See Appendix B
Data	Biodiversity data	Clipping	NA
Data	Biodiversity data	Scaling	Geographical biases were minimized using spThin package, removing occurrences from the same species that are less than 5.5 km from each other
Data	Biodiversity data	Cleaning	Duplicates, records with low-coordinate accuracy, fossil records and entries identified only to the genus-level were removed, further filtering using CoordinateCleaner package, removel of terrestrial occurrences based on geographic polygons from the rnaturalearth package and finally spatial thinning (5.5 km distance) using spThin package
Data	Biodiversity data	Absence data	NA
Data	Biodiversity data	Background data	Accessible but not occupied locations (pixels) were considered as pseudo-absences.
Data	Biodiversity data	Errors and biases	As the data comes from multiple databases, misidentification, potentials, geo-referencing errors and sampling biases cannot be excluded
Data	Data partitioning	Training data	Models were trained and validated using randoms 75/25 splits and five cross-validations
Data	Data partitioning	Validation data	Models were trained and validated using randoms 75/25 splits and five cross-validations
Data	Data partitioning	Test data	No test data
Data	Predictor variables	Predictor variables	Five variables: 5 best marine data layers
Data	Predictor variables	Data sources	Bio-ORACLE (77, 78)
Data	Predictor variables	Spatial extent	-180, 180, -90, 90 (xmin, xmax, ymin, ymax)
Data	Predictor variables	Spatial resolution	~ 5.5 km²
Data	Predictor variables	Coordinate reference system	lon/lat WGS 84 (EPSG:4326)
Data	Predictor variables	Temporal extent	Marine data layers: Decadal from present-day to the end of the 21st century (2000–2100), Occurrences 2000-2024
Data	Predictor variables	Temporal resolution	Mean
Data	Predictor variables	Data processing	No processing
Data	Predictor variables	Errors and biases	NA
Data	Predictor variables	Dimension reduction	To avoid model overfitting, we selected the top five uncorrelated variable (correlation < 0.70)
Data	Transfer data	Data sources	Bio-ORACLE (77, 78)
Data	Transfer data	Spatial extent	-180, 180, -90, 0 (xmin, xmax, ymin, ymax)
Data	Transfer data	Spatial resolution	~ 5.5 km²
Data	Transfer data	Temporal extent	Marine data layers: Decadal from present-day to the end of the 21st century (2000–2100), Occurrences 2000-2024
Data	Transfer data	Temporal resolution	Mean
Data	Transfer data	Models and scenarios	Projection into the current conditions and under three shared socioeconomic pathways (SSP1-2.6, SSP2-4.5 and SSP5-8.5) for the mid- (2040-2050) and long-term (2090-2100)

Data	Transfer data	Data processing	NA
Data	Transfer data	Quantification of Novelty	NA
Model	Variable pre-selection	Variable pre-selection	Five variables: 5 best marine data layers
Model	Multicollinearity	Multicollinearity	Cross-correlations (Pearson)
Model	Model settings	Model settings (fitting)	Default settings for MAXNET and XGBOOST. The other algorithms were optimized using the recommended options with the "Bigboss" pre-defined parameter values for single models.
Model	Model settings	Model settings (extrapolation)	Default settings for MAXNET and XGBOOST. The other algorithms were optimized using the recommended options with the "Bigboss" pre-defined parameter values for single models.
Model	Model estimates	Coefficients	Assessed through 5-fold cross validation
Model	Model estimates	Parameter uncertainty	Assessed through 5-fold cross validation
Model	Model estimates	Variable importance	Built-in the biomod2 package
Model	Model selection - model averaging - ensembles	Model selection	Ensemble models were built from the individual models' weight using a sensitivity metric with a 0.75 threshold TSS
Model	Model selection - model averaging - ensembles	Model averaging	Ensemble models were built from the individual models' weight using a sensitivity metric with a 0.75 threshold TSS
Model	Model selection - model averaging - ensembles	Model ensembles	Ensemble models were built from the individual models' weight using a sensitivity metric with a 0.75 threshold TSS
Model	Analysis and Correction of non-independence	Spatial autocorrelation	Geographical biases were minimized using spThin package for each species with a 5.5 km distance
Model	Analysis and Correction of non-independence	Temporal autocorrelation	NA
Model	Analysis and Correction of non-independence	Nested data	NA
Model	Threshold selection	Threshold selection	0.75 TSS
Assessment	Performance statistics	Performance on training data	AUC and TSS
Assessment	Performance statistics	Performance on validation data	AUC and TSS
Assessment	Performance statistics	Performance on test data	NA
Assessment	Plausibility check	Response shapes	Not assessed
Assessment	Plausibility check	Expert judgement	Yes, plausible results based on other studies and biology of sea turtles
Prediction	Prediction output	Prediction unit	Presence and absence (suitable or not)
Prediction	Prediction output	Post-processing	NA
Prediction	Uncertainty quantification	Algorithmic uncertainty	Assessed through 5-fold cross validation
Prediction	Uncertainty quantification	Input data uncertainty	NA
Prediction	Uncertainty quantification	Parameter uncertainty	Assessed through 5-fold cross validation
Prediction	Uncertainty quantification	Scenario uncertainty	NA
Prediction	Uncertainty quantification	Novel environments	NA

table S3. Summary and sources of variables used in the species distribution models.

Detailed descriptions and additional information are provided in Tables S4 and S5. Note that topographic variables, by definition, lack future projection data.

Type	Abbreviated	Variable name	Unit	Future projection	Number of variables taken in the models	Source
Physical	ocean1	Ocean temperature	°C	Yes	The best five variables	Bio-ORACLE v3.0 (75, 76)
Chemical	ocean2	Salinity	-			
Physical	ocean3	Sea water velocity	m.s-1			
Physical	ocean4	Sea water direction	degree			
Chemical	ocean5	Nitrate	mmol.m-3			
Chemical	ocean6	Phosphate	mmol.m-3			
Chemical	ocean7	Silicate	mmol.m-3			
Chemical	ocean8	Dissolved molecular oxygen	mmol.m-3			
Chemical	ocean9	Iron	mmol.m-3			
Biological	ocean10	Primary productivity	mmol.m-3			
Chemical	ocean11	pH	-			
Biological	ocean12	Chlorophyll	mmol.m-3			
Physical	ocean13	Sea ice thickness	m			
Physical	ocean14	Sea ice cover	Fraction			
Physical	ocean15	Cloud cover	%			
Physical	ocean16	Mixed layer depth	m			
Physical	ocean17	Air temperature	°C			
Topographic	ocean18	Bathymetry	m	No		
	ocean19	Topographic slope	-			
	ocean20	Topographic aspect	-			
	ocean21	Topographic position index	-			
	ocean22	Terrain ruggedness index	-			

table S4. Pearson correlation coefficients between the analyzed variables.

Absolute correlation values exceeding 0.70 are shaded in grey. Detailed descriptions of the variables are provided in Table S3.

	Ocean temperature (Ocean1)	Salinity (Ocean2)	Sea water velocity (Ocean3)	Sea water direction (Ocean4)	Nitrate (Ocean5)	Phosphate (Ocean6)	Silicate (Ocean7)	Dissolved molecular oxygen (Ocean8)	Iron (Ocean9)	Primary productivity (Ocean10)	pH (Ocean11)	Chlorophyll (Ocean12)	Sea ice thickness (Ocean13)	Sea ice cover (Ocean14)	Cloud cover (Ocean15)	Mixed layer depth (Ocean16)	Air temperature (Ocean17)	Bathymetry (Ocean18)	Topographic slope (Ocean19)	Topographic aspect (Ocean20)	Topographic position index (Ocean21)	Terrain ruggedness index (Ocean22)
Ocean1	-	0.470	0.291	0.323	**-0.712**	**-0.755**	-0.598	**-0.980**	-0.079	-0.001	0.052	-0.323	-0.598	-0.689	**-0.904**	-0.283	**0.978**	-0.293	0.083	0.006	-0.001	0.202
Ocean2		-	0.146	0.112	-0.136	-0.218	-0.190	-0.561	-0.479	-0.088	0.269	-0.216	-0.490	-0.489	-0.388	0.203	0.513	-0.372	0.105	0.008	0.001	0.167
Ocean3			-	0.125	-0.049	-0.058	-0.151	-0.278	-0.098	0.126	-0.142	0.066	-0.267	-0.304	-0.283	0.004	0.314	-0.103	0.071	-0.027	-0.001	0.100
Ocean4				-	-0.310	-0.287	-0.115	-0.298	0.060	-0.077	-0.061	-0.212	0.046	0.059	-0.302	-0.306	0.239	0.016	0.002	-0.016	-0.002	0.038
Ocean5					-	**0.968**	**0.842**	0.625	-0.145	0.133	-0.240	0.371	0.131	0.283	0.608	0.468	-0.627	-0.004	-0.007	-0.015	0.002	-0.083
Ocean6						-	**0.813**	0.682	-0.157	0.116	-0.278	0.363	0.235	0.360	0.663	0.409	-0.684	0.027	-0.021	-0.012	0.001	-0.102
Ocean7							-	0.511	0.010	0.071	-0.318	0.244	0.234	0.438	0.473	0.202	-0.579	0.046	-0.031	-0.025	0.000	-0.095
Ocean8								-	0.140	-0.031	-0.064	0.284	0.664	**0.727**	**0.888**	0.189	**-0.973**	0.337	-0.087	-0.005	0.000	-0.207
Ocean9									-	0.300	-0.174	0.270	0.168	0.193	-0.073	-0.294	-0.131	0.547	-0.104	-0.009	-0.009	-0.121
Ocean10										-	-0.099	**0.928**	-0.350	-0.297	-0.166	0.050	0.077	0.258	-0.044	0.004	-0.001	-0.041
Ocean11											-	-0.104	-0.063	-0.103	-0.059	0.108	0.064	0.054	0.042	0.006	-0.001	0.040
Ocean12												-	-0.135	-0.069	0.152	0.195	-0.241	0.300	-0.063	0.004	-0.001	-0.101
Ocean13													-	**0.932**	0.583	-0.241	**-0.729**	0.285	-0.063	0.002	-0.001	-0.155
Ocean14														-	0.644	-0.210	**-0.813**	0.315	-0.078	-0.014	-0.001	-0.177
Ocean15															-	0.300	**-0.891**	0.100	-0.064	0.001	0.003	-0.168
Ocean16																-	-0.181	-0.148	0.019	0.002	0.002	-0.006
Ocean17																	-	-0.329	0.091	0.007	0.000	0.212
Ocean18																		-	-0.022	-0.010	0.106	-0.060
Ocean19																			-	0.000	0.048	**0.930**
Ocean20																				-	0.000	0.001
Ocean21																					-	0.112
Ocean22																						-

table S5. Average importance of each layer in the analysis.

Highlighted values represent the highest importance score with a Pearson correlation below 0.70 (as detailed in Table S4). Species names are abbreviated as follows: Ccar, *Caretta caretta*; Cmyd, *Chelonia mydas*; Dcor, *Dermochelys coriacea*; Eimb, *Eretmochelys imbricata*; Lkem, *Lepidochelys kempii*; Loli, *Lepidochelys olivacea*; Ndep, *Natator depressus*

Abbreviated	Variable	Ccar	Cmyd	Dcor	Eimb	Lkem	Loli	Ndep
ocean1	Ocean temperature	0.216	**0.136**	0.116	0.112	0.171	**0.194**	0.138
ocean2	Salinity	**0.103**	0.016	**0.047**	0.012	0.020	0.026	0.069
ocean3	Sea water velocity	0.017	0.007	0.012	0.008	0.009	0.006	0.010
ocean4	Sea water direction	0.018	0.009	0.010	0.006	0.016	0.006	0.019
ocean5	Nitrate	0.054	0.045	0.067	**0.055**	0.083	0.081	**0.199**
ocean6	Phosphate	**0.207**	0.053	**0.158**	0.046	**0.255**	0.048	0.075
ocean7	Silicate	0.055	0.035	0.051	0.043	0.077	0.051	0.107
ocean8	Dissolved molecular oxygen	**0.239**	0.129	0.130	**0.155**	0.149	0.126	**0.224**
ocean9	Iron	0.062	**0.058**	**0.082**	**0.085**	**0.134**	**0.069**	**0.218**
ocean10	Primary productivity	**0.089**	**0.074**	0.047	0.024	**0.176**	**0.100**	0.085
ocean11	pH	**0.066**	0.017	**0.047**	0.012	0.021	0.013	0.041
ocean12	Chlorophyll	0.075	0.037	0.046	0.017	0.129	0.026	**0.089**
ocean13	Sea ice thickness	0.017	**0.047**	0.040	**0.043**	0.047	**0.072**	0.060
ocean14	Sea ice cover	0.034	0.032	0.031	0.067	0.058	0.051	0.054
ocean15	Cloud cover	0.224	0.132	0.043	0.047	0.108	0.076	0.211
ocean16	Mixed layer depth	0.040	**0.043**	0.025	**0.055**	**0.045**	**0.173**	0.075
ocean17	Air temperature	0.137	0.124	**0.213**	0.099	**0.232**	0.167	0.146
ocean18	Bathymetry	**0.073**	**0.319**	**0.200**	**0.487**	**0.083**	**0.161**	**0.544**
ocean19	Topographic slope	0.008	0.015	0.008	0.010	0.013	0.005	0.082
ocean20	Topographic aspect	0.011	0.009	0.011	0.009	0.018	0.011	0.012
ocean21	Topographic position index	0.010	0.009	0.009	0.010	0.026	0.008	0.065
ocean22	Terrain ruggedness index	0.013	0.021	0.012	0.024	0.022	0.006	**0.105**

table S6. Changes in potential distribution for each species under various scenarios compared to the current distribution. Metrics include decrease, increase and range shift (calculated as increase minus decrease). Values with a range shift exceeding 50% are in bold. Negative range shifts are displayed in red, positive shifts in blue and near-zero shifts (-5% to 5%) in grey. Species abbreviations: Ccar, *Caretta caretta*; Cmyd, *Chelonia mydas*; Dcor, *Dermochelys coriacea*; Eimb, *Eretmochelys imbricata*; Lkem, *Lepidochelys kempii*; Loli, *Lepidochelys olivacea*; Ndep, *Natator depressus*

Evaluation metric	Algorithm	Ccar	Cmyd	Dcor	Eimb	Lkem	Loli	Ndep
SSP1-2.6 2050	Decrease	56.11%	11.48%	37.80%	2.28%	42.24%	27.83%	2.22%
	Increase	6.58%	50.64%	36.57%	22.81%	14.81%	5.66%	24.73%
	Range change	-49.53%	39.16%	-1.23%	20.53%	-27.43%	-22.17%	22.50%
SSP2-4.5 2050	Decrease	65.01%	6.79%	30.32%	1.42%	26.79%	24.08%	3.51%
	Increase	4.40%	79.33%	37.29%	28.58%	23.57%	23.20%	18.29%
	Range change	**-60.61%**	**72.54%**	6.96%	27.16%	-3.23%	-0.89%	14.78%
SSP5-8.5 2050	Decrease	75.00%	8.29%	46.27%	0.89%	18.79%	20.32%	6.05%
	Increase	2.44%	8.33%	12.31%	7.31%	13.00%	8.28%	11.42%
	Range change	**-72.56%**	0.04%	-33.96%	6.42%	-5.79%	-12.04%	5.36%
SSP1-2.6 2100	Decrease	56.99%	12.28%	39.39%	2.35%	50.80%	30.73%	4.35%
	Increase	11.29%	45.26%	30.34%	25.00%	15.46%	9.04%	34.02%
	Range change	-45.70%	32.98%	-9.05%	22.65%	-35.34%	-21.69%	29.67%
SSP2-4.5 2100	Decrease	83.39%	15.13%	-54.96%	1.74%	60.73%	43.85%	8.26%
	Increase	0.69%	67.74%	30.07%	36.84%	12.23%	16.06%	37.48%
	Range change	**-82.70%**	**52.60%**	-24.89%	35.10%	-48.50%	-27.79%	29.22%
SSP5-8.5 2100	Decrease	90.53%	17.34%	66.05%	0.59%	37.62%	48.14%	13.66%
	Increase	1.20%	21.88%	24.42%	25.25%	34.49%	22.68%	50.52%
	Range change	**-89.33%**	4.55%	-41.63%	24.66%	-3.13%	-25.46%	36.86%
Average of all scenarios		**-67%**	**34%**	**-17%**	**23%**	**-21%**	**-18%**	**23%**

table S7. Evaluation metrics for each species and algorithm, including the consensus model (weighted mean ± standard deviation) from species distribution models.

Metrics reported: Receiver Operating Characteristic (ROC) and True Skill Statistic (TSS). Species abbreviations: Ccar, *Caretta caretta*; Cmyd, *Chelonia mydas*; Dcor, *Dermochelys coriacea*; Eimb, *Eretmochelys imbricata*; Lkem, *Lepidochelys kempii*; Loli, *Lepidochelys olivacea*; Ndep, *Natator depressus*.

Evaluation metric	Algorithm	Ccar	Cmyd	Dcor	Eimb	Lkem	Loli	Ndep
ROC (AUC)	ANN	0.934 ± 0.015	0.960 ± 0.003	0.963 ± 0.002	0.982 ± 0.002	0.993 ± 0.001	0.982 ± 0.002	0.996 ± 0.002
	CTA	0.965 ± 0.004	0.969 ± 0.005	0.974 ± 0.004	0.970 ± 0.004	0.990 ± 0.003	0.978 ± 0.004	0.990 ± 0.004
	GAM	0.954 ± 0.002	0.973 ± 0.001	0.976 ± 0.001	0.983 ± 0.001	0.996 ± 0.001	0.986 ± 0.001	0.999 ± 0.001
	GBM	0.972 ± 0.001	0.980 ± 0.001	0.980 ± 0.001	0.988 ± 0.001	0.997 ± 0.001	0.990 ± 0.001	0.999 ± 0.001
	GLM	0.916 ± 0.002	0.963 ± 0.001	0.960 ± 0.001	0.982 ± 0.001	0.994 ± 0.001	0.974 ± 0.029	0.996 ± 0.006
	MARS	0.943 ± 0.004	0.970 ± 0.001	0.967 ± 0.002	0.982 ± 0.002	0.995 ± 0.001	0.984 ± 0.001	0.998 ± 0.002
	MAXNET	0.933 ± 0.002	0.966 ± 0.002	0.969 ± 0.001	0.983 ± 0.001	0.995 ± 0.001	0.985 ± 0.001	0.998 ± 0.001
	RF	1.000	1.000	1.000	1.000	1.000	1.000	1.000
	SRE	0.796 ± 0.004	0.841 ± 0.002	0.778 ± 0.003	0.888 ± 0.002	0.889 ± 0.002	0.884 ± 0.003	0.896 ± 0.009
	XGBOOST	1.000	1.000	1.000	1.000	1.000	1.000	1.000
	Ensemble (Wmean)	0.989	0.988	0.990	0.993	0.999	0.994	1.000
TSS	ANN	0.792 ± 0.034	0.831 ± 0.010	0.805 ± 0.014	0.910 ± 0.004	0.958 ± 0.004	0.901 ± 0.007	0.983 ± 0.005
	CTA	0.876 ± 0.011	0.907 ± 0.005	0.904 ± 0.010	0.925 ± 0.008	0.968 ± 0.005	0.933 ± 0.008	0.979 ± 0.010
	GAM	0.813 ± 0.004	0.870 ± 0.004	0.858 ± 0.006	0.914 ± 0.005	0.968 ± 0.003	0.923 ± 0.007	0.995 ± 0.004
	GBM	0.847 ± 0.003	0.899 ± 0.005	0.871 ± 0.005	0.929 ± 0.004	0.969 ± 0.003	0.935 ± 0.005	0.993 ± 0.004
	GLM	0.724 ± 0.007	0.835 ± 0.003	0.781 ± 0.013	0.912 ± 0.005	0.955 ± 0.002	0.888 ± 0.037	0.979 ± 0.011
	MARS	0.774 ± 0.014	0.848 ± 0.009	0.829 ± 0.016	0.910 ± 0.004	0.961 ± 0.003	0.908 ± 0.006	0.983 ± 0.008
	MAXNET	0.750 ± 0.010	0.819 ± 0.005	0.818 ± 0.003	0.912 ± 0.004	0.956 ± 0.002	0.907 ± 0.007	0.980 ± 0.005
	RF	0.997 ± 0.001	0.990 ± 0.001	0.990 ± 0.002	0.985 ± 0.002	0.996 ± 0.001	0.991 ± 0.002	1.000
	SRE	0.593 ± 0.009	0.681 ± 0.004	0.556 ± 0.006	0.777 ± 0.005	0.778 ± 0.004	0.768 ± 0.005	0.791 ± 0.018
	XGBOOST	0.995 ± 0.005	0.992 ± 0.006	0.999 ± 0.003	0.999 ± 0.002	1.000	1.000	1.000
	Ensemble (Wmean)	0.908	0.909	0.903	0.926	0.972	0.939	0.987

table S8. Contribution of environmental variables to SDMs for each species.

Species abbreviations: Ccar, *Caretta caretta*; Cmyd, *Chelonia mydas*; Dcor, *Dermochelys coriacea*; Eimb, *Eretmochelys imbricata*; Lkem, *Lepidochelys kempii*; Loli, *Lepidochelys olivacea*; Ndep, *Natator depressus*

Abbreviated	Variable	Ccar	Cmyd	Dcor	Eimb	Lkem	Loli	Ndep
ocean1	Ocean temperature		0.329				0.301	
ocean2	Salinity	0.212		0.065				
ocean3	Sea water velocity							
ocean4	Sea water direction							
ocean5	Nitrate				0.122			0.345
ocean6	Phosphate	0.322		0.134		0.311		
ocean7	Silicate							
ocean8	Dissolved molecular oxygen	0.254			0.347			0.217
ocean9	Iron		0.107	0.160	0.066	0.155	0.087	0.125
ocean10	Primary productivity	0.310	0.044			0.367	0.136	
ocean11	pH	0.055		0.045				
ocean12	Chlorophyll							0.095
ocean13	Sea ice thickness		0.064		0.037		0.051	
ocean14	Sea ice cover							
ocean15	Cloud cover							
ocean16	Mixed layer depth		0.056		0.052	0.034	0.218	
ocean17	Air temperature			0.337		0.239		
ocean18	Bathymetry	0.107	0.361	0.287	0.512	0.042	0.156	0.692
ocean19	Topographic slope							
ocean20	Topographic aspect							
ocean21	Topographic position index							
ocean22	Terrain ruggedness index							0.111

table S9. Hotspot area (km²) and percentage of hotspots under protection or unprotected for current conditions and SSP5-8.5 projections for 2050 and 2100.

Region	Current			SSP5-8.5 2050			SSP5-8.5 2100		
	Hotspot surface (km^2)	% Unprotected	% Protected	Hotspot surface (km^2)	% Unprotected	% Protected	Hotspot surface (km^2)	% Unprotected	% Protected
Arctic Ocean	0	-	-	0	-	-	0	-	-
Baltic Sea	0	-	-	0	-	-	0	-	-
Indian Ocean	365,810	78.7	21.3	103,096	61.4	38.6	102,388	61.0	39.0
Mediterranean Region	42,045	96.3	3.7	36,717	91.1	8.9	133,652	84.1	15.9
North Atlantic Ocean	766,243	88.6	11.4	340,799	93.7	6.3	171,447	95.6	4.4
North Pacific Ocean	36,008	73.8	26.2	26,305	53.2	46.8	72,756	57.5	42.5
South Atlantic Ocean	173,295	75.4	24.6	126,198	81.0	19.0	106,515	84.9	15.1
South China and Eastern Archipelagic Seas	210,997	97.7	2.3	103,004	99.2	0.8	6284	100.0	0
South Pacific Ocean	220,608	16.8	83.2	148,714	15.4	84.6	100,478	59.5	40.5
Southern Ocean	0	-	-	0	-	-	0	-	-
